# Video dataset of Balinese dance basic movement for action recognition

**DOI:** 10.1016/j.dib.2024.110189

**Published:** 2024-02-13

**Authors:** I Nyoman Rudy Hendrawan, Putu Setyarini, Putu Andika Tedja Permana, I Made Hermanto, I Gusti Agung Putu Dharma Putra, Anak Agung Ayu Citra Maharani

**Affiliations:** Information System, Department of Informatics and Computer, Institut Teknologi dan Bisnis STIKOM Bali, Denpasar, Indonesia

**Keywords:** Video dataset, Balinese dance, Action recognition

## Abstract

In the convergence of cultural heritage preservation and computational research, this paper presents a comprehensive dataset capturing the nuanced artistry of traditional Balinese dancing. This endeavour not only documents but also aims to protect these intricate dance movements, bridging technological advancements with rich cultural traditions. The dataset includes 1740 and 828 high-definition video recordings of female and male dancers, respectively, showcasing 24 unique dance movements. These recordings, made using multiple smartphone cameras from various perspectives, reflect the dance's complex nature and emphasize gender-based movement variations, key for detailed cultural analysis. Our systematic approach involved three strategically placed cameras, capturing diverse angles for a holistic view. The subsequent preprocessing stage, including segmenting and labelling, has enhanced the dataset's clarity, making it a valuable resource for cultural studies and computational analysis in preserving intangible cultural heritage. The videos, stored in MP4 format, are categorized by dancer gender, dance type, and camera angle, offering researchers a rich, multifaceted tool for exploring this traditional art form.

Specifications Table

**Table:** 

Subject	Computer Visions and Pattern Recognition
Specific subject area	Video dataset of basic Balinese dance movement.
Data format	Raw
Type of data	MP4
Data collection	Our dataset was meticulously assembled over two distinct sessions, capturing an array of traditional Balinese dance movements using various smartphone cameras from three different angles. In the first session, we focused on thirteen fundamental female dance movements: *Ngelung, Ngeseh, Tapak Sirangpada, Ngeed, Ngelo, Ngumbang, Agem Kanan, Agem Kiri, Ulap-Ulap, Ngegol, Seledet, Nyalud*, and *Nyeregseg*. These were performed by nine skilled female dancers, reflecting the movements' gender-specific nature. The second session concentrated on eleven male dance movements: *Agem Kanan, Agem Kiri, Gandang-Gandang, Malpal, Nayog, Nepuk Kampuh, Oyod, Piles, Seledet, Tapak Sirangpada*, and *Ulap-Ulap*, showcasing the diversity and richness of male dance traditions. Following the data capture, we embarked on a meticulous preprocessing stage. This involved manually cutting and labeling each video segment to ensure precision and ease of use in future analyses. This process was crucial for maintaining the integrity and clarity of the dance movements, thereby facilitating accurate cultural documentation and analysis.
Data source location	Institution: Institut Teknologi dan Bisnis STIKOM Bali City/Town/Region: Denpasar, Bali Country: Indonesia
Data accessibility	Repository name: Mendeley Data Data identification number: (1) DOI: 10.17632/s2gv9d6gpb.2; (2) DOI: 10.17632/tr9py7hzj4.1 Direct URL to data: https://data.mendeley.com/datasets/s2gv9d6gpb/2 https://data.mendeley.com/datasets/tr9py7hzj4/1

## 1 Value of the Data

• Cultural Preservation and Education: This dataset is a vital tool for preserving Bali's rich cultural heritage, offering a digital archive of traditional Balinese dance movements. It serves as a comprehensive educational resource for students, researchers, and practitioners, enabling in-depth study and appreciation of these dance forms, thereby ensuring their longevity and continued relevance.
• Dance Training and Choreography: Dance instructors and students can use this dataset as a detailed reference for practicing and mastering Balinese dance movements. Choreographers can also explore this resource to innovate, blending traditional movements with contemporary styles, or to create entirely new dance routines, fostering creative expression within and beyond the Balinese dance community.
• Human Activity Recognition and Computer Vision: This dataset is invaluable for researchers and developers in human activity recognition and computer vision. It provides a unique opportunity to develop and refine machine learning models aimed at understanding dance movements. Applications stemming from this research could range from virtual dance trainers to augmented reality experiences and intuitive gesture-based interfaces.
• Cross-cultural Comparison: By offering a detailed view of Balinese dance movements, this dataset opens avenues for comparative studies with other dance forms and cultures. Such analysis can shed light on the nuances of movement patterns, styles, and techniques, contributing to a richer understanding of global dance traditions and fostering cross-cultural appreciation and exchange.

## 2 Background

The primary objective of generating this dataset is to meticulously document and preserve the fundamental movements of Balinese dance, a cornerstone of Balinese cultural heritage. In doing so, this dataset emerges as a multifaceted resource serving not only in cultural preservation but also as a foundation for educational and research endeavours [1,2]. It offers an in-depth analysis of traditional Balinese dance forms, enabling extensive cross-cultural comparisons with other global dance styles. Furthermore, this dataset is poised to significantly contribute to the fields of machine learning and computer vision, particularly in human activity recognition.

Beyond serving as a valuable tool for dance practitioners and cultural researchers, this dataset aims to engage a wider audience, including developers and the public. It provides a robust platform for exploration, learning, and the creation of new experiences inspired by Balinese dance movements [3], thereby fostering a deeper global appreciation of this rich cultural art form. The dataset also sets the stage for future interdisciplinary research and development, bridging cultural heritage with cutting-edge technology, and expanding the horizons of both traditional and modern fields of study [4].

## 3 Data Description

The raw video footage was segmented into distinct sections, each corresponding to specific dance movements and performers. This process resulted in a compilation of 1740 video files for the female dance dataset (Table 1), the snapshot of the sample video shown in Table 2. For the male dance dataset, the limited availability of performers resulted in a smaller collection of 828 video files (Table 3), the snapshot of the sample video shown in Table 4. The video data, stored in a high-definition format, is structured to facilitate easy access and analysis [5,6]. It is intended to be a valuable resource for researchers, educators, and practitioners interested in traditional Balinese dance, as well as those in the fields of human activity recognition and computer vision.

**Table 1:** Number of female dance video per class.

Num.	Data Class	Number of Data
1.	Agem Kanan	120
2.	Agem Kiri	120
3.	Ngegol	120
4.	Nyalud	120
5.	Nyeregseg	120
6.	Seledet	120
7.	Ulap-Ulap	120
8.	Ngeed	150
9.	Ngelo	150
10.	Ngelung	150
11.	Ngeseh	150
12.	Ngumbang	150
13.	Tapak Sirangpada	150

**Table 2:** Snapshots of video samples in the female dance video.

Movement	Example of Video Data		
	Left Camera	Middle Camera	Right Camera
Ngelung	Snapshot time at 00:01	Snapshot time at 00:01	Snapshot time at 00:02
Ngeseh	Snapshot time at 00:02	Snapshot time at 00:03	Snapshot time at 00:02
Tapak Sirangpada	Snapshot time at 00:01	Snapshot time at 00:01	Snapshot time at 00:01
Ngeed	Snapshot time at 00:02	Snapshot time at 00:02	Snapshot time at 00:02
Ngelo	Snapshot time at 00:03	Snapshot time at 00:04	Snapshot time at 00:05
Ngumbang	Snapshot time at 00:01	Snapshot time at 00:04	Snapshot time at 00:03
Agem Kanan	Snapshot time at 00:03	Snapshot time at 00:02	Snapshot time at 00:02
Agem Kiri	Snapshot time at 00:02	Snapshot time at 00:01	Snapshot time at 00:03
Ngegol	Snapshot time at 00:02	Snapshot time at 00:03	Snapshot time at 00:02
Nyalud	Snapshot time at 00:02	Snapshot time at 00:02	Snapshot time at 00:02
Nyeregseg	Snapshot time at 00:02	Snapshot time at 00:06	Snapshot time at 00:03
Seledet	Snapshot time at 00:01	Snapshot time at 00:00	Snapshot time at 00:01
Ulap-Ulap	Snapshot time at 00:03	Snapshot time at 00:02	Snapshot time at 00:04

**Table 3:** Number of male dance video per class.

Num.	Data Class	Number of Data
1.	Agem Kanan	75
2.	Agem Kiri	75
3.	Gandang-Gandang	76
4.	Malpal	77
5.	Nayog	75
6.	Nepuk Kampuh	75
7.	Oyod	75
8.	Piles	75
9.	Seledet	75
10.	Tapak Sirang Pada	75
11.	Ulap-Ulap	75

**Table 4:** Snapshots of video samples in the male dance video.

Movement	Example of Video Data		
	Left Camera	Middle Camera	Right Camera
Agem Kanan	Snapshot time at 00:02	Snapshot time at 00:03	Snapshot time at 00:01
Agem Kiri	Snapshot time at 00:01	Snapshot time at 00:01	Snapshot time at 00:01
Gandang Gandang	Snapshot time at 00:03	Snapshot time at 00:03	Snapshot time at 00:03
Malpal	Snapshot time at 00:04	Snapshot time at 00:02	Snapshot time at 00:02
Nayog	Snapshot time at 00:08	napshot time at 00:06	Snapshot time at 00:02
Nepuk Kampung	Snapshot time at 00:02	Snapshot time at 00:03	Snapshot time at 00:02
Oyod	Snapshot time at 00:03	Snapshot time at 00:03	Snapshot time at 00:03
Piles	Snapshot time at 00:01	Snapshot time at 00:01	Snapshot time at 00:02
Seledet	Snapshot time at 00:01	Snapshot time at 00:01	Snapshot time at 00:01
Tapak Sirang Pada	Snapshot time at 00:05	Snapshot time at 00:05	Snapshot time at 00:01
Ulap-Ulap	Snapshot time at 00:02	Snapshot time at 00:01	Snapshot time at 00:04

**Fig 1:**
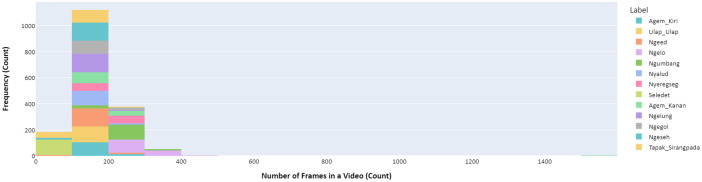
Number of frames distribution for female dance video dataset.

**Fig 2:**
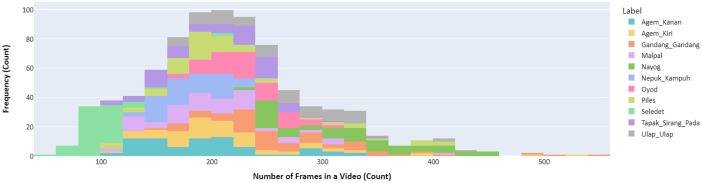
Number of frames distribution for male dance video dataset.

For detailed insights into the dataset's video frame characteristics, refer to Table 5, Figs. 1, and 2. Each video is predominantly recorded at a frame rate of 30 frames per second (fps). This consistency in frame rate was maintained to ensure uniformity in data quality, which is particularly beneficial for computational analysis and machine learning applications where frame rate consistency can be crucial. However, there are some instances where videos have slightly less than 30 fps. These variations are minimal and do not significantly deviate from the 30 fps standard, thus maintaining the overall integrity and usability of the dataset for most applications (as detailed in Table 6).

**Table 5:** Number of frames data description.

Attributes	Female Dance Video	Male Dance Video
Count	1740	828
Mean	166.7	217.3
Std. Dev	67.9	77.8
Min.	47	55
Percentile 25%	127	165.8
Percentile 50%	156	207
Percentile 75%	199	256
Max.	1564	546

**Table 6:** Number of frames per second data description.

Attributes	Female Dance Video	Male Dance Video
Count	1740	828
Mean	30	30.13
Std. Dev	0.03	0.07
Min.	29.97	30.03
Percentile 25%	29.98	30.09
Percentile 50%	30	30.12
Percentile 75%	30	30.15
Max.	30.27	30.53

Regarding frame size, an interesting variation is observed. The female dance video data encompasses two different frame sizes. This variation was a result of the diverse camera specifications used during the recording sessions. In contrast, the male dance video dataset maintains a consistent frame size across all videos. While this discrepancy in frame size between the two datasets could present challenges in certain types of analysis, it also offers an opportunity for testing the robustness of video processing and analysis algorithms in handling varied frame sizes.

The implications of these technical details are significant for potential users of the dataset. Those engaged in fields like human activity recognition, computer vision, and cultural studies must take these variations into account when designing their experiments or applications. The dataset provides a unique opportunity to explore and develop methodologies that are adaptable to variations in video data specifications.

## 4 Experimental Design, Materials and Methods

The dataset was meticulously compiled over two distinct sessions, with the first focusing on capturing dance movements performed by female dancers and the second on male dancers. This gender-specific approach was integral to accurately documenting the unique aspects of each dance style. We invited all Balinese dancers affiliated with our institution to participate in the data gathering phase according to a predetermined schedule. Unfortunately, not all dancers were able to participate in the procedure.

In total, 13 basic dance movements for female dancers (detailed in Table 1) and 11 for male dancers (Table 3) were recorded. The first session saw the participation of nine female performers in individual video recordings, while the second session involved five male performers. Each session employed a tri-camera setup, strategically placing cameras on the right, left, and front sides to capture a comprehensive view of the dance movements (examples of the video capture are showcased in Tables 2 and 4). This setup ensured a multi-dimensional representation of each dance movement.

For the video recording of female dancers, a tri-camera setup was utilized to capture a range of angles. From the left side, a Samsung Galaxy S8 was employed, featuring a 12-megapixel single lens. The front perspective was recorded using a Xiaomi Redmi Note 9, with its primary 48-megapixel lens. The right side was documented with an Oppo F1s, which has a 13-megapixel single lens. The male dancers were recorded using a different set of devices. The left angle was captured with an iPhone XR, which includes a 12-megapixel lens. The central angle was covered by the Samsung Galaxy S22 Ultra, with a 108-megapixel lens. Lastly, the right-side footage was taken using a Xiaomi Redmi Note 9, with 48-megapixel lens.

The data is meticulously organized into three directories for ease of use in various applications: training data, validation data, and test data. Accompanying these directories are annotation files in CSV format (train.csv, val.csv, and test.csv), which include essential details like file names, labels, and file paths. These annotations play a crucial role in preventing data leakage during experimental setups or model training. In terms of distribution, the female dancer video dataset comprises 1115 training videos, 277 validation videos, and 348 test videos. Conversely, the male dancer video dataset includes 531 training videos, 132 validation videos, and 165 test videos. This distribution was determined by allocating 80% of the total data to the training set and 20% to the test set. From the training set, 20% was further segregated to form the validation set. This structure, while standard, is flexible and can be reconfigured based on specific experimental requirements. Users can merge and redistribute the data by adjusting the proportions in the annotation files as necessary for their research or application.

The preprocessing stage of this dataset is pivotal, especially for applications involving deep learning classification. Users should familiarize themselves with the characteristics of the video data, including the number of frames, frame rate, and frame size. Tools like Python library OpenCV and Pandas can be instrumental in acquiring this information. The implementation of the experimental design of this dataset can be accessed at our code repository [7] (Table 7).

**Table 7:** Frame size data description.

Dataset	Frame Size (width, length)	Number
Female Dance Video	(1920, 1080)	900
	(1280, 720)	840
Male Dance Video	(1920, 1080)	828

## Limitations

The dataset possesses several significant limitations that could potentially impact its utility and analysis. One of the major obstacles encountered was the limited availability of highly skilled dancers to join the study, resulting in a lack of diversity and thoroughness in the recorded dance moves. Furthermore, the scarcity of male dancers results in a comparatively smaller number of male dancer recordings than female ones. Another notable issue within the male dancer video dataset is the presence of overly extended videos. This is attributed to inaccuracies in the video cutting process, leading to the inclusion of extraneous frames that do not contribute valuable information regarding the dance movements. Such limitations underscore the need for a more rigorous data collection and processing methodology, particularly in securing a larger and more skilled pool of dancers and in ensuring precision in video editing to maintain the dataset's relevance and utility.

## Ethics Statement

The study complied with ethical standards by obtaining participants' informed consent, which explicitly included their agreement to the display of their faces in the dataset, and the author further implemented measures to safeguard the confidentiality of the participants.

## CRediT authorship contribution statement

**I Nyoman Rudy Hendrawan:** Conceptualization, Software, Validation, Funding acquisition, Methodology, Writing – review & editing. **Putu Setyarini:** Conceptualization, Data curation, Supervision. **Putu Andika Tedja Permana:** Writing – original draft, Visualization, Investigation. **I Made Hermanto:** . **I Gusti Agung Putu Dharma Putra:** Data curation, Software, Resources. **Anak Agung Ayu Citra Maharani:** Data curation, Resources.

## Data Availability

Video Dataset of Male Basic Balinese Dance Movement for Action Recognition (Original data) (Mendeley Data) Video Dataset of Woman Basic Balinese Dance Movement for Action Recognition (Original data) (Mendeley Data)
